# Kurt Schapira MD FRCP FRCPsych DPM: Formerly Consultant Psychiatrist, Newcastle Health Authority, Honorary Lecturer in Psychiatry, University of Newcastle

**DOI:** 10.1192/pb.bp.117.056093

**Published:** 2017-10

**Authors:** Kenneth Davison, Hamish McClelland, Alan Kerr, Martin Schapira

**Figure:**
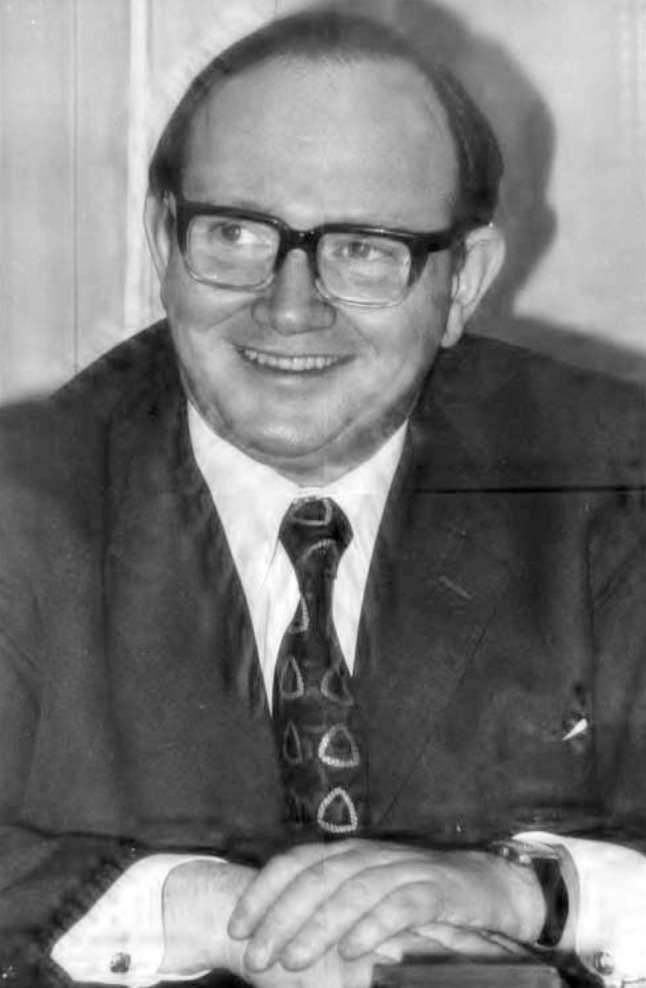


Shortly after completing his house officer posts and national service in the Royal Army Medical Corps, Kurt Schapira, who died recently aged 87, was appointed a research fellow to the eminent neurologist Henry Miller. More than 1150 patients with multiple sclerosis resident in Northumberland and Durham on 1 January 1958 were reviewed by Dr Schapira. Using this material, he jointly wrote 16 scientific articles on various aspects of multiple sclerosis – including its epidemiology and clinical features – many of which are still cited today. The study made a major contribution to knowledge of this condition.^1^ Subsequently, fascinated by the emotional effects of multiple sclerosis on the patients, Dr Schapira decided to switch to psychiatry. He participated in Martin Roth's landmark studies on anxiety and depression and undertook his own research into suicide and attempted suicide, as well as on the treatment of anorexia nervosa. His longitudinal studies relating suicide rates to social factors and the availability of lethal methods were influential in demonstrating the importance of a public health preventive approach to suicide.^2^ He published several articles on these and other topics, including, with others, a much-quoted paper on the influence of tablet colour in the treatment of anxiety and depressive states.^3^

Born in Vienna to orthodox Jewish parents, Kurt's childhood was disrupted by the death of his mother when he was aged 7 years. This was followed by the incarceration of his father by the Nazis in Dachau and then Buchenwald concentration camps. Fortunately, his father was released from Buchenwald and allowed to travel to England before the Second World War. Kurt lived in Berlin with a maternal aunt until the age of 10, when he obtained a place on a Kindertransport train to England. Having successfully negotiated a path through the British educational system, Kurt was admitted to medical school in Newcastle – then part of the University of Durham – and qualified as a doctor in 1952.

He trained in psychiatry in the university department of psychiatry in Newcastle, headed at that time by Martin Roth and James Gibbons. Within the department he was promoted to first assistant and then senior lecturer. In 1974 he became consultant psychiatrist to the Newcastle Health Authority while retaining his university connection as an honorary lecturer. He continued in this position until his retirement from the NHS in 1989.

He lectured widely both in the UK and abroad, as far afield as Brisbane, Minnesota, Montreal and Jerusalem. During his retirement he remained active in research, especially into factors influencing the incidence of suicide in Newcastle over three decades. His last published article on the subject^4^ – in collaboration with others, including his son Martin – appeared as recently as June 2016.

He undertook many responsibilities in both National Health Service and university administration and was a member of several university committees. Within the Royal College of Psychiatrists, he was a member of Council (1979–82), the Education Committee (1979–82) and the Board of Assessors of the *British Journal of Psychiatry* (1970–75), as well as examiner for the membership and various other psychiatry-related degrees at home and abroad. He was also elected President of the Section of Psychiatry, Royal Society of Medicine (1980) and President of the North of England Medico-Legal Society (1982).

Kurt enjoyed teaching and regularly topped the students' ratings for the quality of his lectures, which were notable for their combination of wit and erudition. These attributes rendered him a popular speaker to non-medical audiences, especially on the art of Chagall, Salvador Dali and Van Gogh, and the artistic portrayal of doctors and lawyers. He was a connoisseur of opera and classical music, particularly Verdi and Mozart.

He was generous with his time in acceding to invitations to speak about his childhood experiences to a variety of audiences, from schoolchildren to prisoners, and was proud to meet Prince Charles recently at a reception for Kindertransport survivors. A genial extrovert and renowned raconteur, whose personality raised the spirits of his friends and colleagues, he had a fund of humorous anecdotes, enhanced in the telling by a trace of a Viennese accent. He often expressed his indebtedness to England for accepting him as a refugee. Considering his many contributions to the sum of human health and happiness, any debt has been more than repaid.

He leaves Eva, his wife of 51 years, 3 children, 5 grandchildren, 4 great grandchildren and an older sister.
